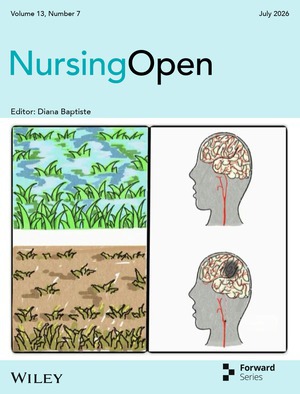# Cover Article

**DOI:** 10.1002/nop2.70681

**Published:** 2026-07-28

**Authors:** Jie Yu, Rong Xiao, Jiru Liu, Fang Tian, Bei Wu, Juan Li

## Abstract

The cover image is based on the article *Influence of Decision‐Makers' Perceived Risk on the Propensity for Intravenous Thrombolysis in Acute Ischemic Stroke: A Cross‐Sectional Study* by Jie Yu et al., https://doi.org/10.1002/nop2.70513.